# Following Enzyme Activity with Infrared Spectroscopy

**DOI:** 10.3390/s100402626

**Published:** 2010-03-25

**Authors:** Saroj Kumar, Andreas Barth

**Affiliations:** Department of Biochemistry and Biophysics, Stockholm University, Stockholm, Sweden; E-Mail: saroj@dbb.su.se

**Keywords:** vibrational spectroscopy, infrared spectroscopy, ATR, FTIR, enzyme activity

## Abstract

Fourier transform infrared (FTIR) spectroscopy provides a direct, “on-line” monitor of enzymatic reactions. Measurement of enzymatic activity is based on the fact that the infrared spectra of reactants and products of an enzymatic reaction are usually different. Several examples are given using the enzymes pyruvate kinase, fumarase and alcohol dehydrogenase. The main advantage of the infrared method is that it observes the reaction of interest directly, *i.e.,* no activity assay is required to convert the progress of the reaction into an observable quantity.

## 1. Introduction

Enzymes are fundamental to life and are used in many biotechnological processes. Therefore, enzymatic activity is an important parameter in many contexts. In favourable cases an enzymatic reaction can be directly followed with spectroscopy in the UV or visible spectral range. In these cases the substrate or product of an enzymatic reaction is coloured, like for the NAD^+^ / NADH system, or light is emitted in the course of the reaction, like in the reaction of luciferase with ATP and luciferin.

In most cases, however, substrate and product cannot be distinguished in the UV or visible range of the spectrum and enzyme activity has to be determined indirectly. For this, coloured or fluorescent substrate analogues have been developed or the enzymatic reaction of interest has to be coupled to auxiliary enzymatic reactions that can be followed in the UV or visible spectral range. An example is the coupled enzyme assay for the measurement of ATPase activity. The assay couples ATP hydrolysis catalysed by the enzyme of interest to the oxidation of NADH via the auxiliary enzymes pyruvate kinase and lactate dehydrogenase.

This “state of the art” of activity measurements implies that for a “new” enzymatic reaction an activity assay or a suitable substrate analogue has to be developed. This is often time-consuming, the modification of the substrate may affect the activity and applying the activity assay can be cumbersome, slow and may be limited to specific experimental conditions required by the assay. Therefore, in order to speed up the development of biotechnological processes and to save costs, techniques that monitor enzymatic reactions directly are highly desirable.

Infrared spectroscopy can provide such a direct, “on-line” monitor of enzymatic reactions, because the spectrum depends on the structure and environment of a molecule. When a molecular structure is modified in an enzymatic reaction, the infrared spectrum is altered and changes in infrared absorption can be followed to monitor the progress of the reaction. Measurements of enzyme activity with infrared spectroscopy are relatively straightforward and several studies have been published comprising urea hydrolysis by urease [1,2], cefoxitin hydrolysis by β-lactamase [3], deacylation of cinnamoyl-chymotrypsin [4], ATP hydrolysis by the Ca^2+^-ATPase [5–7], dephosphorylation of fructose 1,6-bisphosphate by fructose-1,6 bisphosphatase [8] and of 4-nitrophenylphosphate by alkaline phosphatase [9], oxidation of d-glucose by glucose oxidase [10], hydrolysis of sucrose by β-fructofuranosidase [11–13], of maltose by amyloglucosidase [13] and of starch by amylogucosidase [14,15] and α-amylase [15,16], hydrolysis of amides [17,18] and synthesis of hydroxamic acid derivatives [18] by amidase, hydrolysis of several organophosphorus compounds by diisopropyl fluorophosphatase [19], the reaction of α-ketoglutarate and Ala to Glu and pyruvate by glutamic-pyruvic transaminase [20], consumption of oxalate and production of formate and CO_2_ by oxalate decarboxylase [21], and acetone-butanol fermentation [22].

We have previously followed ATP hydrolysis by the Ca^2+^-ATPase with infrared spectroscopy [5–7]. Enzyme activity could be measured with only 7.5 μg enzyme being needed and the infrared activity values compared well to those of the traditional coupled enzyme assay. In these measurements, the enzymatic reaction was started by the photolytic release of ATP from a biologically inactive photosensitive precursor molecule-caged ATP [5,6]. Because this method is not generally applicable with commercial equipment, we explore here a simpler approach to follow enzymatic activity. Using a commercial attenuated total reflection (ATR) setup, we monitored the enzymatic reactions of pyruvate kinase, alcohol dehydrogenase and fumarase.

Pyruvate kinase (PK) (EC 2.7.1.40) is a key enzyme of the glycolytic pathway that catalyses the transfer of phosphate from phosphoenolpyruvate (PEP) to adenosine diphosphate (ADP). The physiological reaction of PK proceeds in two chemical steps. The first step is phosphoryl transfer from PEP to ADP which produces ATP and the enolate of pyruvate [23]. The second step is the addition of a proton to the enolate of pyruvate to produce pyruvate [24] (Scheme I).

Fumarase (EC 4.2.1.2) is an enzyme of the tricarboxylic acid (Krebs) cycle which reversibly catalyses the conversion from malate to fumarate [25] (Scheme II).

Alcohol dehydrogenase (ADH) (EC 1.1.1.1) is a member of a general class of enzymes called oxidoreductases that facilitate the interconversion of alcohols to aldehydes. The reactions need the coenzyme nicotinamide adenine dinucleotide (NAD^+^) (Scheme III).

## 2. Results and Discussion

### 2.1. Experimental Approach

Enzyme activity was monitored in the following way: enzyme and substrate were manually mixed and placed on an ATR crystal. Then a background spectrum and a series of sample spectra were recorded. Each of the difference spectra shown in the following reflects the difference in absorbance between a particular sample spectrum and the background spectrum, *i.e.*, the absorbance change that occurred in the time between recording background and sample spectrum. In this way the much stronger absorptions of water, buffer and protein do not contribute to the difference spectra shown, as long as they remain constant. Buffer signals in the difference spectra are expected, if the catalytic reaction involves proton uptake or release. Small protein signals were observed, because protein settled on the surface of the ATR crystal. This was however outside the spectral ranges evaluated for monitoring the enzymes.

### 2.2. Infrared Difference Spectra of the Catalytic Reaction of PK

**Figure 1.**
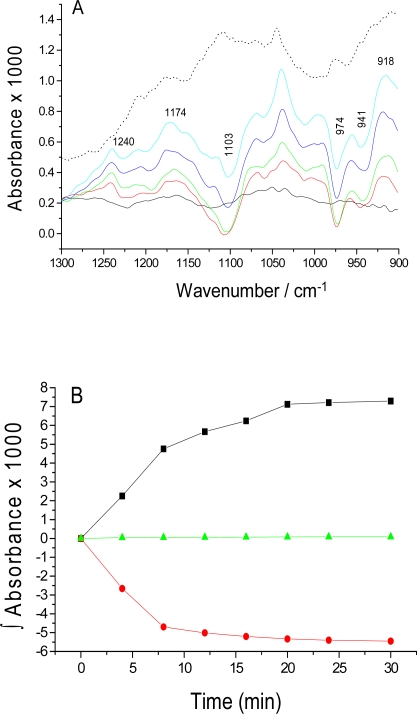
Enzymatic reaction of PK. **(A)** Series of overlaid spectra (solid lines) of infrared absorbance changes upon PEP and ADP addition to PK, observed for 30 min. The solid line spectra were recorded at 12 s (black), 4 min (red), 8 min (green), 16 min (blue) and 30 min (cyan). The dotted line is the last spectrum (30 min) of the control experiments (shifted up). **(B)** Kinetics of the enzymatic reaction of PK, monitored by integrated band intensities at 1240 cm^−1^ (black) and 941 cm^−1^ (red) and the control experiment (941 cm^−1^, green). Time zero is the time when recording of the first spectrum after the background spectrum started.

The absorbance changes due to the catalytic reaction of PK are shown in Figure 1. They represent the difference in absorbance between the background spectrum recorded within 50 s after mixing and spectra recorded at later times. Negative bands are due to substrate consumption and positive bands due to product formation. The series of solid line spectra in Figure 1A represent the catalytic reaction during 30 minutes and the dotted line is the last spectrum of the control experiments. The positive band at 1174 cm^−1^ is due to the formation of pyruvate [26], and the negative bands at 974 cm^−1^ and 1103 cm^−1^ are caused by PEP consumption [27]. Similarly, absorption at 1240, and 918 cm^−1^ is assigned to ATP production while the negative band at 941 cm^−1^ is due to ADP consumption [28,29]. These bands are assigned as follows: the band at 1174 cm^−1^ is due to the C-CH_3_ stretching vibration of pyruvate [30], and the bands at 1103 and 974 cm^−1^ are due to -PO_3_^2−^ stretching vibrations of PEP [27]. The bands arising at 1240 cm^−1^ and 918 cm^−1^ are from the β-PO_2_^−^ and the P-O-P groups of ATP, respectively, while the β-PO_3_^2−^ and P-O-P groups of ADP are observed at 941 cm^−1^ [28,29,31]. These bands are not observed in control experiments without ADP. However, the baseline is not completely flat due to the small amplitude of the signals and the long measurement time. The kinetic evolution of ATP production (1240 cm^−1^, black) and ADP consumption (941 cm^−1^, red) is shown in Figure 1B while the control (941 cm^−1^, green) sample, containing only PK and PEP, did not show any such changes. Similar plots could also be obtained for the mentioned bands of PEP and pyruvate (data not shown).

### 2.3. Infrared Spectra of the Catalytic Reaction of Fumarase

**Figure 2.**
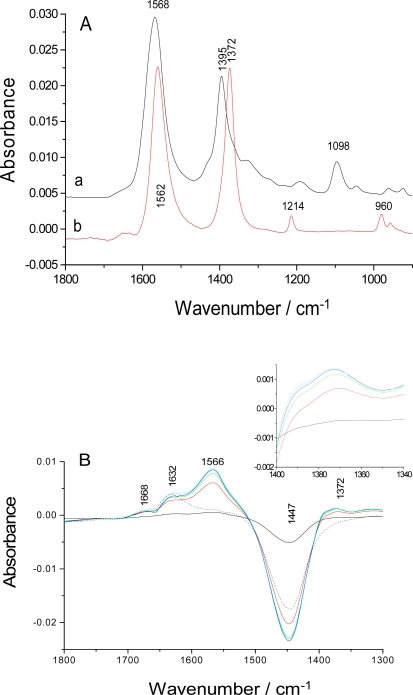

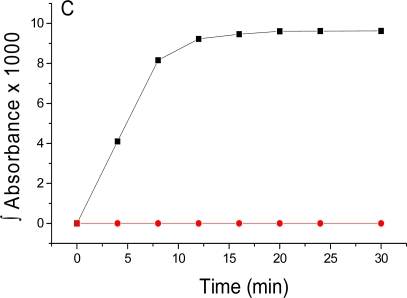
**(A)** Infrared spectra of 100 mM malate (trace a) and fumarate (trace b) in H_2_O at pH 7.5. **(B)** Series of overlaid spectra of infrared absorbance changes due to the malic acid to fumarate reaction. The solid line spectra were recorded at 12 s (black), 4 min (red), 8 min (green), 16 min (blue) and 30 min (cyan) and the dotted line spectrum is the last spectrum of the control experiments without malic acid (30 min). The insert in panel B is an expanded view of the 1372 cm^−1^ band. **(C)** Kinetics of the enzymatic reaction of fumarase, monitored by the integrated band intensity at 1372 cm^−1^ for reaction (black) and the control (red) experiments.

Fumarase is an enzyme which reversibly catalyses the interconversion between malate and fumarate. Figure 2A shows the absorbance spectra of malic acid (a) and fumaric acid (b) at pH 7.5.

The absorption band at 1568 cm^−1^ in the malic acid spectrum and that at 1562 cm^−1^ in the fumaric acid spectrum are attributed to the COO^−^ antisymmetric stretching vibration. The bands of the symmetric stretching vibration were observed at 1395 and 1372 cm^−1^ respectively [30]. The band at 1214 cm^−1^ in fumaric acid is attributed to the C-O (H) stretching vibration [30]. Figure 2B shows successive difference spectra of the catalytic reaction of fumarase during 30 minutes. The initial mixture contains the substrate malic acid and enzyme fumarase at pH 7.5. As the reaction proceeds, one observes the rise of the 1372 cm^−1^ band of fumaric acid. The insert shows the evolution of the 1372 cm^−1^ band on an expanded scale. The control spectrum after 30 min, obtained without malic acid, is shown as dotted line. For the kinetic evaluation we selected the 1372 cm^−1^ band because this band was not overlapped (see control) with any other band. The kinetics of the 1372 cm^−1^ band is shown in Figure 2C and reflects the formation of the product. The equilibrium of the reaction was reached approximately within 12 minutes. The control did not show a signal at this wavenumber.

### 2.4. Infrared Difference Spectra of the Catalytic Reaction of ADH

**Figure 3.**
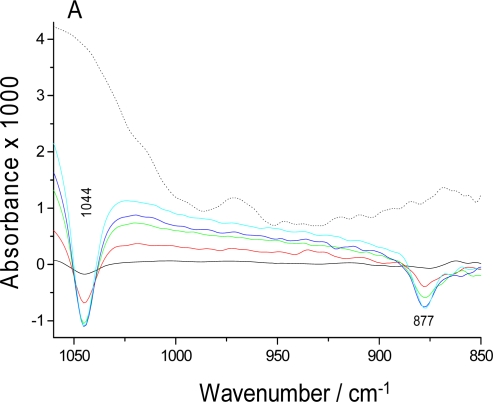

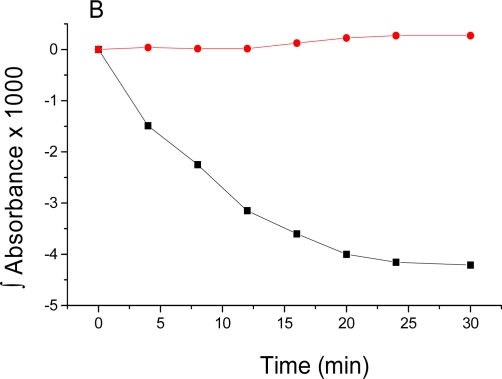
Enzymatic reaction of ADH. **(A)** Series of overlaid spectra (solid line) of infrared absorbance changes upon ethanol addition to ADH, recorded for 30 minutes. The spectra in solid line were recorded at 12 s (black), 4 min (red), 8 min (green), 16 min (blue) and 30 min (cyan). The dotted line is the last spectrum (30 min) of the control experiments without substrate. **(B)** Kinetics of the enzymatic reaction of ADH monitored by the integrated band intensity at 877 cm^−1^ for reaction (black) and the control (red) experiments.

Alcohol dehydrogenase catalyses the interconversion between ethanol and acetaldehyde. The difference spectra of ethanol consumption and acetaldehyde formation are shown in Figure 3A. They were taken during 30 minutes after mixing enzyme and substrate. Negative bands at 1044 and 877 cm^−1^ indicate consumption of ethanol (solid lines) and are due to the C-O stretching and C-C stretching vibrations of ethanol respectively [32]. We also observed the rising of negative bands at 2981 and 2900 cm^−1^ from CH stretching vibrations (not shown in Figure 3). The control experiment (dotted line) with a sample of ADH without substrate did not show these bands. Figure 3B shows a plot of the kinetics of this reaction using the 877 cm^−1^ band. The equilibrium was reached approximately within 24 minutes. Again, the control did not show a signal.

## 3. Experimental Procedures

### 3.1. Materials

PK from rabbit muscle, ADH from *Saccharomyces cerevisiae*, fumarase from porcine heart, monopotassium salt of PEP, pyruvate, ADP, NADH^+^ (nicotinamide adenine dinucleotide reduced), MOPS (3-[*N*-morpholino]propanesulphonic acid) and potassium phosphate monobasic were purchased from Sigma. Tris-HCl was obtained from Angus. Potassium chloride (KCl), zinc chloride (ZnCl_2_) and magnesium chloride MgCl_2_) were obtained from Scharlau.

### 3.2. Protein Sample Preparation

For PK: 0.21 mM (15 units/μL) rabbit muscle PK was prepared in buffer (50 mM Tris-HCl + MOPS, pH 7.5) containing 100 mM KCl and 5 mM of MgCl_2_. 100 mM of PEP and ADP were dissolved in the above buffer and the pH adjusted to 7.5.

For fumarase: 0.034 mM porcine heart fumarase was prepared in buffer (50 mM Tris-HCl + MOPS, pH 7.5) containing 100 mM KCl and 5 mM of MgCl_2._ Malic acid 100 mM was dissolved in the above buffer.

For ADH: 0.35 mM (15 units/μL) *Saccharomyces cerevisiae* ADH were prepared in buffer (50 mM Tris-HCl + MOPS, pH 8.8) containing 100 mM KCl and 5 mM ZnCl_2_. Ethanol (200 mM) was dissolved in the above buffer.

### 3.3. FTIR Studies

FTIR spectra were recorded at 4 cm^−1^ resolution on a Bruker Vertex 70 FTIR spectrometer equipped with an HgCdTe detector using a SensIR ATR setup for liquid samples with a diamond reflection element.

For PK: 5 μL PK, 8 μL ADP and 7 μL of PEP from the above stock solutions were mixed in a vial, 20 μL of mixed sample was placed on the ATR diamond reflection element and the sample trough closed with a lid to avoid evaporation of the sample. The final concentrations were as follows: 0.052 mM (60 μg per measurement) PK, 25 mM ADP (85 μg) and 25 mM PEP (35.5 μg). Then, a 300 scan single beam spectrum (background spectrum) and repeated spectra in the absorption mode (150 scans each) were recorded for 30 minutes. It took around 50 s to prepare the reaction mixture (15 s) and to measure the background spectrum (35 s), this was followed by recording of the first spectrum (12 s) and spectra recording for further 30 minutes. The control samples contained PK and PEP in the same above concentrations but no ADP and the measurements were performed in the same way as for the reaction mixture. We averaged four samples for activity measurement and three samples for the controls.

For fumarase: 15 μL fumarase, 5 μL malic acid from the above stock solutions were mixed in a vial and 20 μL of mixed sample was placed on the ATR diamond reflection element. The final concentrations of the reaction mixture were 0.0255 mM fumarase (74.2 μg per measurement) and 25 mM malic acid (17 μg). The control samples contained the same amount of fumarase but no malic acid. Spectra were recorded as stated above. We averaged four samples for activity measurement and three samples for the controls. For recording absorption spectra, 100 mM malic acid and fumaric acid were dissolved in water and the pH was adjusted to 7.5.

For ADH: 1 μL ADH and 19 μL ethanol (200 mM) from the above stock solutions were mixed in a vial and 20 μL of mixed sample was placed on the ATR diamond reflection element. The final concentrations of the reaction mixture were 0.0175 mM ADH (3.5 μg per measurements) and 190 mM ethanol (175 μg). The control samples contained 0.0175 mM (3.5 μg) of ADH. Spectra were recorded as stated above. We averaged four samples for activity measurement and three samples for the controls.

For the kinetic evaluation of particular bands in a series of difference spectra, the band was integrated around its maximum with respect to a baseline drawn through two data points on both sides of the maximum. Each of these points was the average of several data points near the base of the band. The spectral range for the baseline points was broad when there was no overlap with other bands, it was narrow and closer to the position of the band maximum when other bands were superimposing. This results in integrated band areas which are highly specific for the band studied and little affected by baseline drifts or overlap with other bands.

## 4. Conclusions

This work demonstrates the benefits of infrared spectroscopy for monitoring enzymatic reactions. Substrates and products can be observed directly without the need of time-consuming assay development. Here we studied enzyme activity of PK, fumarase and ADH using commercial equipment. For all three enzymes, we followed their activity by observing either reactant consumption or product formation or both (phosphoenolpyruvate, ADP, ethanol and fumaric acid) by using a particular infrared absorption band of these molecules. Kinetic data were obtained by using only ≤1 nmol enzyme (1 nmol PK, 0.5 nmol fumarase, 0.35 nmol ADH) and ∼μmol substrate (0.5 μmol PEP, ADP, and malic acid and 4 μmol ethanol). If quantitative activity measurements are necessary, the signal amplitudes of the kinetic experiments can easily be expressed in absolute concentrations by comparison with absorption spectra of reactants and/or products at known concentrations.

## Figures and Tables

**Scheme I.**
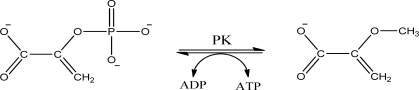
Conversion of PEP to pyruvate by PK.

**Scheme II.**
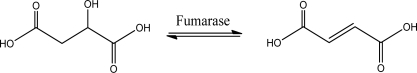
Conversion of malic acid to fumaric acid by fumarase.

**Scheme III.**
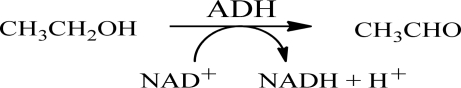
Conversion of ethanol to acetaldehyde by alcohol dehydrogenase.

## Abbreviations

ATR: attenuated total reflection
FTIR: Fourier transformed infrared
IR: infrared
PEP: phosphoenolpyruvate
PK: pyruvate kinase
ADH: alcohol dehydrogenase
